# Enhanced surveillance for severe pneumonia, Thailand 2010–2015

**DOI:** 10.1186/s12889-019-6774-5

**Published:** 2019-05-10

**Authors:** Charatdao Bunthi, Henry C. Baggett, Christopher J. Gregory, Somsak Thamthitiwat, Thitipong Yingyong, Wantana Paveenkittiporn, Anusak Kerdsin, Malinee Chittaganpitch, Ruchira Ruangchira-urai, Pasakorn Akarasewi, Kumnuan Ungchusak

**Affiliations:** 1 Division of Global Health Protection, Thailand Ministry of Public Health–US Centers for Disease Control and Prevention Collaboration, Tivanond Road, Nonthaburi, 11000 Thailand; 2 Department of Disease Control, Bureau of Epidemiology, Ministry of Public Health, Tivanond Road, Nonthaburi, 11000 Thailand; 3 National Institute of Health, Ministry of Public Health, Tivanond Road, Nonthaburi, 11000 Thailand; 4 Faculty of Public Health, Kasetsart University Chalermphrakiat Sakon Nakhon Province Campus, Sakon Nakhon, 47000 Thailand; 5 Siriraj Hospital, Mahidol University, Bangkok, Thailand; 6 Division of Global Health Protection, Centers for Disease Control and Prevention, Clifton Road, Atlanta, GA 30329 USA

**Keywords:** Community-acquired pneumonia, CAP, Severe pneumonia, Surveillance, Global health security

## Abstract

**Background:**

The etiology of severe pneumonia is frequently not identified by routine disease surveillance in Thailand. Since 2010, the Thailand Ministry of Public Health (MOPH) and US CDC have conducted surveillance to detect known and new etiologies of severe pneumonia.

**Methods:**

Surveillance for severe community-acquired pneumonia was initiated in December 2010 among 30 hospitals in 17 provinces covering all regions of Thailand. Interlinked clinical, laboratory, pathological and epidemiological components of the network were created with specialized guidelines for each to aid case investigation and notification. Severe pneumonia was defined as chest-radiograph confirmed pneumonia of unknown etiology in a patient hospitalized ≤48 h and requiring intubation with ventilator support or who died within 48 h after hospitalization; patients with underlying chronic pulmonary or neurological disease were excluded. Respiratory and pathological specimens were tested by reverse transcription polymerase chain reaction for nine viruses, including Middle East Respiratory Syndrome Coronavirus (MERS-CoV), and 14 bacteria. Cases were reported via a secure web-based system.

**Results:**

Of specimens from 972 cases available for testing during December 2010 through December 2015, 589 (60.6%) had a potential etiology identified; 399 (67.8%) were from children aged < 5 years. At least one viral agent was detected in 394 (40.5%) cases, with the most common of single vial pathogen detected being respiratory syncytial virus (RSV) (110/589, 18.7%) especially in children under 5 years. Bacterial pathogens were detected in 341 cases of which 67 cases had apparent mixed infections. The system added MERS-CoV testing in September 2012 as part of Thailand’s outbreak preparedness; no cases were identified from the 767 samples tested.

**Conclusions:**

Enhanced surveillance improved the understanding of the etiology of severe pneumonia cases and improved the MOPH’s preparedness and response capacity for emerging respiratory pathogens in Thailand thereby enhanced global health security. Guidelines for investigation of severe pneumonia from this project were incorporated into surveillance and research activities within Thailand and shared for adaption by other countries.

## Background

Emerging or re-emerging infections, including avian influenza (AI), pandemic human influenza and coronaviruses such as severe acute respiratory syndrome (SARS) and Middle East respiratory syndrome (MERS-CoV), can cause severe respiratory illness and death and cause international outbreaks that can threaten global health security [1–6]. In 2003 and 2004, the World Health Organization reported that the avian influenza A H5N1 virus had spread from Asia to Europe and Africa, resulting in millions of poultry infections, 50 human cases, and 36 human deaths [7, 8]. These avian influenza outbreaks have had serious impact on national economies and international trade. In response, the Thailand Ministry of Public Health (MOPH) established the National Avian Influenza Surveillance (NAIS) system in 2004 to detect influenza in severe pneumonia patients [9]. However, due to limited resources, the NAIS did not conduct diagnostic testing for other pathogens in severe pneumonia cases that tested negative for influenza. To expand this influenza-specific system, an enhanced surveillance system for severe and fatal pneumonia (SevPn) was established by the Bureau of Epidemiology (BOE) and National Institute of Health (NIH), Thailand MOPH, in collaboration with the United States Centers for Disease Control and Prevention (US CDC).

On December 1, 2010, the SevPn surveillance network began among 30 public hospitals in 17 provinces in Thailand with the following objectives: identify potential pathogens causing severe pneumonia; create networks to develop standardized guidelines for case investigation; provide clinical consultation or diagnostic services; and create a database of severe pneumonia cases and a specimen bank of samples from these cases for potential future testing when new methods are available. Findings from this surveillance system was intended to provide information that lead to some policy changes or recommendation on pneumonia case management guidelines in the future.

This report describes the methodology of the SevPn surveillance system and provides preliminary results on each of its objectives from December 2010 through December 2015.

## Methods

### Surveillance sites

**Fig. 1:**
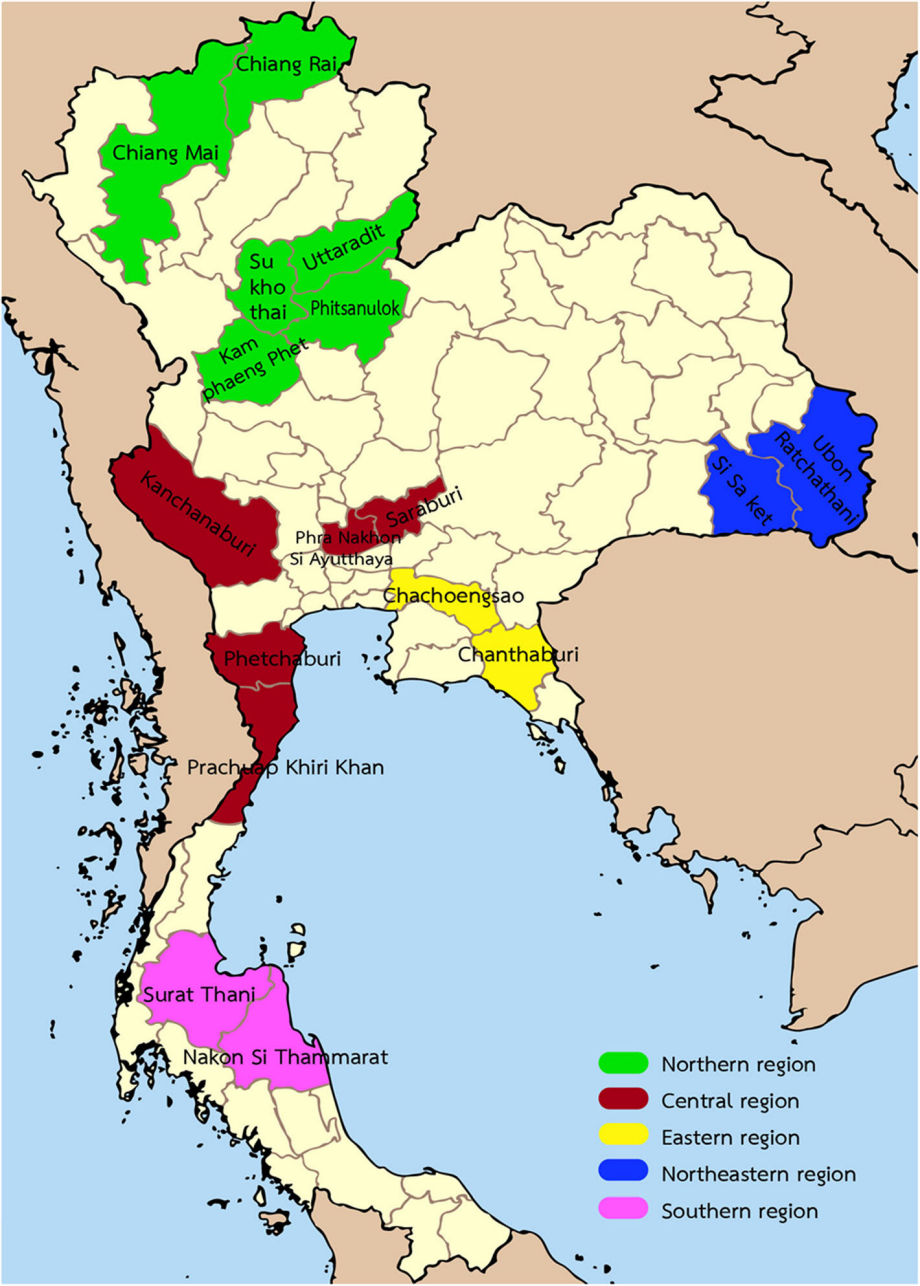
Author created a regional map of the site locations for severe pneumonia enhanced surveillance network in Thailand from 2010 to 2015

Thailand is a middle to high income country with the estimated population of 69 million in 2016. The population density was 135 people per square kilometer and varies from 100 to 250 people per square kilometer in each region [10]. Surveillance was conducted in 17 provinces in all five regions of Thailand (Fig. 1). The 30 participating hospitals in these provinces were selected based on having an intensive care unit and hospital staff willing to participate in the SevPn activities.

### Surveillance system structure

#### Clinical network

This network, established by BOE, comprised experts in clinical infectious diseases, virology, bacteriology, radiology, pathology and epidemiology. The network, based in Bangkok with members from MOPH and various academic centers, developed the severe pneumonia case definition, notification and investigation guidelines for SevPn surveillance. The expert members of this network served as technical consultants for physicians and nurses at the surveillance hospitals who identified cases and provided treatment. The focal person was the project data officer at BOE, but physicians were able to contact the experts directly or through the BOE.

#### Laboratory network

The laboratory network provided laboratory testing and consultation to the clinicians of the surveillance hospitals and transferred specimens to other laboratories as needed. The focal point was a staff member at the specimen receiving and distributing center of NIH.

#### Pathology network

The pathology network comprised medical and veterinary pathologists from academic centers and the Thailand Zoological Park Organization and developed post-mortem examination guidelines. These guidelines instructed non-pathologists in performing percutaneous transthoracic lung needle biopsy to collect appropriate and adequate lung tissue in fatal pneumonia cases. The network provided consultations on autopsy and histopathology testing through the pathology focal points, which were the four regional pathology network hubs located in the academic institutions in each of these regions; in the North, Northeast, South and Central regions of Thailand.

#### Epidemiology network

This network operated through epidemiologists in the 30 surveillance hospitals who also served as members of ‘Surveillance and Rapid Response Teams’ (SRRT) nationwide. The SRRT is a national system of epidemiology and investigation teams from the central through community level established in 2004 to rapidly detect and respond to emerging public health threats. The SRRTs performed disease surveillance for outbreaks, conducted field investigations and implemented necessary responses [11]. The hospital epidemiologist performed case notification and investigation at the hospitals where SevPn cases were identified. There were also SRRT epidemiologists at the district, provincial, and community levels who worked closely with the hospital SRRT on case investigations for severe pneumonia cases, particularly for cases with contact with sick persons or sick or dead poultry or other animals, or with a history of travel. The focal person for the epidemiology network was the project manager at the BOE.

### Surveillance case definition

A severe pneumonia case was defined as community-acquired pneumonia (CAP) with radiographic findings consistent with pneumonia as determined by clinician or radiologist, no etiology was identified by laboratory testing available at the hospital and in a patient aged ≥2 months, requiring ventilator support and hospitalized ≤48 h. Cases also included patients with CAP who died without being ventilated and within 48 h of admission. Patients were excluded if illness onset occurred ≥2 weeks before identification or if they had been hospitalized within the prior week. Patients were also excluded if they had known hospital-acquired pneumonia, chronic pulmonary disease (chronic obstructive pulmonary disease, chronic bronchitis, chronic bronchiectasis or pulmonary dysplasia in children), swallowing dysfunction, or a neurological condition causing inability to perform daily activities.

### Case finding and ascertainment

At each surveillance hospital, a designated focal person for the SevPn network ensured that the guidelines developed by the clinical network were followed. The focal persons, hospital physicians and nurses, were trained by BOE project staff on the surveillance case definition, exclusion criteria, data entry systems, and specimen collection and handling, including post mortem transthoracic needle biopsy. On a daily basis, each hospital focal person screened patients admitted to the intensive care unit to assess if they met the SevPn case definition.

### Collection of clinical information and specimens

Demographic, clinical, epidemiological and hospital laboratory information were collected from each patient’s medical record by the surveillance site focal person using a standard case report form and entered into an online reporting system. Data were updated when the patient was discharged from the hospital. In addition to routine specimens collected for clinical care, including blood culture, clinicians collected tracheal aspirates (intubated patients), acute serum and convalescent serum at 2 weeks later if possible. Nasopharyngeal or throat swabs were collected in patients who died without intubation. In fatal cases, Transthoracic cardiac puncture was encouraged to collect heart blood if serum was not available; if consent was provided, lung tissue specimens were collected by transthoracic needle biopsy [12] and deposited in two sterile tubes and a separate container with 10% formalin. All specimens were kept cold and sent to NIH within 24 h.

### Laboratory testing

Respiratory, blood, and serum specimens were shipped to NIH in Bangkok where they were stored and tested or sent to other academic centers that had laboratory capacity for further testing based on clinician request. For the tracheal aspirates, viral and bacterial testing were performed on different samples. Tracheal aspirates in viral transport media (VTM) were tested by multiplex real-time reverse transcription polymerase chain reaction (rRT-PCR) [13] for a panel of six viruses including influenza A and B, parainfluenza virus, adenovirus, respiratory syncytial virus (RSV), and human metapneumovirus. Singleplex rRT-PCR was performed for subtyping of influenza A-positive specimens and for MERS Co-V starting in September 2012 [14]. For bacterial testing, tracheal aspirates in sterile tubes (without VTM) were tested by conventional multiplex PCR for 11 bacterial pathogens: *Streptococcus pneumoniae, Haemophilus influenzae, Moraxella catarrhalis, Klebsiella pneumoniae, Pseudomonas aeruginosa, Acinetobacter baumannii, Stenotrophomonas maltophilia, Escherichia coli, Staphylococcus aureus, Burkholderia pseudomallei* and *Acinetobacter* species. A separate multiplex real-time PCR was used to detect atypical bacteria including *Mycoplasma pneumoniae, Chlamydophila pneumoniae,* and *Legionella* species*.* All multiplex PCRs used for testing were developed by the NIH [15].

Lung tissue from fatal cases were sent to the regional pathology network for histopathological testing where they were embedded in paraffin, cut into 3 μm-thick sections, deparaffinized in xylene, and rehydrated in graded alcohol. Each section was stained with hematoxylin and eosin. Additional specialized testing was performed if there was clinical suspicion for a particular disease, such as silver staining for *Pneumocystis jirovecii*. Acute serum and/or convalescent serum were sent for serological testing and storage for further diagnostic testing if required by the clinical committees.

### Case reporting and monitoring

The case reporting system at BOE and the laboratory testing system at NIH were activated when a focal person at a surveillance hospital entered the patient’s information into the online reporting system. After the specimens arrived and were tested at NIH, a focal person at the laboratory entered the test result in the online system.

**Fig. 2:**
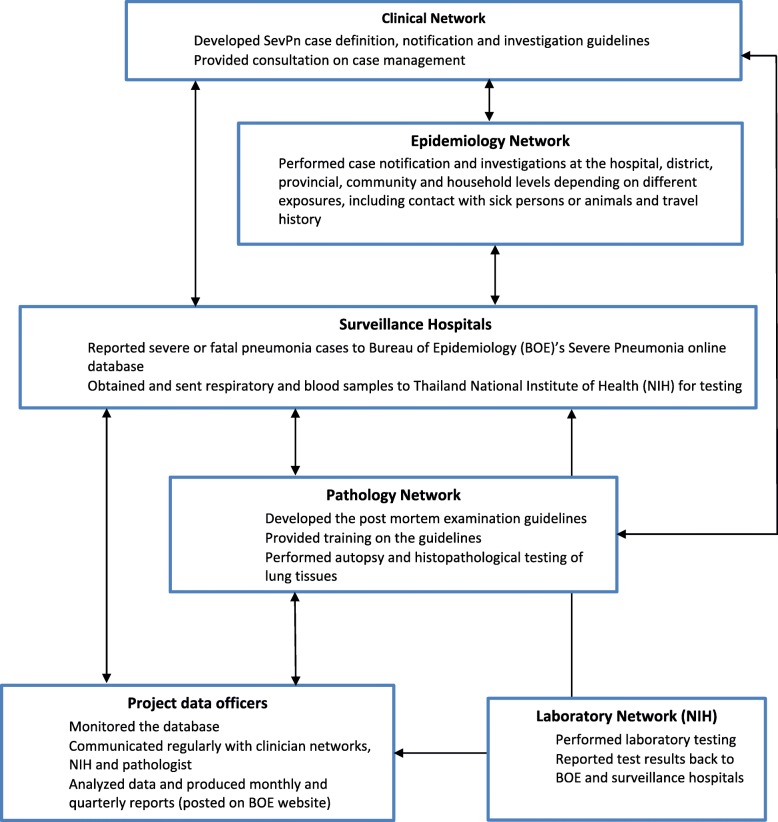
Structure of severe pneumonia enhanced surveillance network

At BOE, the project data officer routinely checked the data on the severe pneumonia web-based system and followed up with the hospital focal person as needed to ensure the accuracy and completeness of the report. A report was considered complete when all fields of the online case record form were completed. Laboratory results were made available to the attending physicians in each hospital via a password protected online database. BOE posted monthly, quarterly and annual severe and fatal pneumonia surveillance reports on the BOE website that were available to the public (Fig. 2). Each hospital was able to export their data and conduct hospital-level reports themselves.

Medical and laboratory records from all cases were reviewed and presented in a quarterly clinical network meeting to confirm that reported cases met the case definition and, through a case review process, assign the likely causative pathogen. The expert committee also suggested additional testing that might be necessary for a final etiologic determination.

### Surveillance audit

SevPn surveillance staff conducted two surveillance audits in all 30 hospitals during the 4 years of project implementation. The first audit was conducted during 2011–2012 and the second during 2013–2014. BOE staff, including physicians, nurses and data officers, visited each hospital. They reviewed the medical records of all hospitalized patients who required mechanical ventilation and had been discharged with International Classification of Diseases (ICD-10) codes consistent with a diagnosis of pneumonia including codes J12 to J16 and J18 and who were admitted during the selected 3-month audit period. Once potential cases were identified, all medical records were reviewed to determine whether they met the surveillance case definition and had been reported to the SevPn system.

### Data analysis

The sensitivity of the SevPn system was calculated by dividing the number of cases reported to the surveillance system that met the SevPn case definition by the total number of eligible cases (number of cases meeting the SevPn case definition from chart review audit). Descriptive data are presented as frequencies for discrete variables, and mean or median for continuous variables. SPSS version 18.0 was used for all analyses.

### Ethical review

The study proposal was reviewed and determined to be a routine public health activity for public health surveillance by Thailand MOPH Ethical Review Committee. Confidentiality was maintained by using a password protected online system that limited access to authorized persons.

## Results

### Demographic data

A total of 972 patients met the case definition of severe pneumonia of unknown etiology, including 220 (22.6%) fatal cases, and were reported to the BOE severe pneumonia system during December 2010 through December 2015 (Table 1). Of these, 580 (59.7%) were male. The majority (61.7%) were children aged < 5 years and the largest proportion (35.1%) of cases came from the southern region of Thailand. Of 220 fatal cases, lung biopsy was performed in 13 (5.9%).

**Table 1:** Demographic characteristics of patients enrolled in the severe pneumonia enhanced surveillance network, Thailand 2010–2015

Characteristics	Fatal cases (%)	Non-fatal cases (%)	Overall (%)
Reported pneumonia cases	*N* = 220	*N* = 752	*N* = 972
Male	134 (60.9)	446 (59.3)	580 (59.7)
Female	86 (39.1)	306 (40.7)	392 (40.3)
Region			
North	74 (33.6)	215 (28.6)	289 (29.7)
Northeast	10 (4.6)	14 (1.9)	24 (2.5)
East	18 (8.2)	59 (7.8)	77 (7.9)
Central	92 (41.8)	149 (19.8)	241 (24.8)
South	26 (11.8)	315 (42.9)	341 (35.1)
Age group (Year)			
< 5	60 (27.3)	540 (71.8)	600 (61.7)
5–9	7 (3.2)	28 (3.7)	35 (3.6)
10–19	10 (4.5)	18 (2.4)	28 (2.9)
20–39	33 (15.)	35 (4.7)	68 (7.0)
40–60	42 (19.1)	53 (7.0)	95 (9.8)
> 60	68 (30.9)	78 (10.4)	146 (15.0)

### Types of specimen

Tracheal secretion was the main specimen tested for viruses (946, 99.4%) and bacteria (823, 99%) while five cases had lung tissues tested for bacterial and four cases for viruses. Only two NP swabs were sent for viral and bacterial testing. Twenty cases had no specimen for viral testing and 141 cases had no specimen for bacterial testing either because the hospital did not sent the specimen or the specimens were not adequate for testing. Blood cultures were done at the participating hospitals per their routine practice and only 337 patients (34.7%) had results available.

### Etiologic identification

All reported cases had at least one specimen submitted for testing. Among respiratory specimens from all 972 cases, 589 (60.6%) tested positive for at least one potential pathogen (Table 2). A virus was detected in 394 (40.5%) of cases with 236 (24.3%) of cases having a single virus as the only detected pathogen. RSV was the most commonly detected virus overall (12.3%), followed by influenza A and influenza B (3.9%), and adenovirus (3.0%). A bacteria was detected in 341 (35%) of cases with 128 (13.2%) of cases, having a single bacteria as the only detected pathogen. *M. pneumoniae* was the most commonly detected bacteria overall (4.2%), followed by *H. influenzae* (1.7%), *M. catarrhalis* (1.4%) and *S. pneumoniae* (1.0%). Mixed detection were found in 225 cases (23.1%).

**Table 2:** Pathogens detected through enhanced surveillance for severe pneumonia, by patient outcome, Thailand 2010–2015

Findings	Age < 5 years			Age > 5 years		
	Fatal cases (%)	Non-fatal cases (%)	Overall (%)	Fatal cases (%)	Non-fatal cases (%)	Overall (%)
Reported cases	60 (6.2)	540 (55.5)	600 (61.7)	160 (16.5)	212 (21.8)	372 (38.3)
Cases with positive laboratory results	31 (5.3)	368 (62.5)	399 (67.7)	94 (16)	96 (6.3)	190 (32.3)
Virus^a^	17 (3)	167 (28.4)	184 (31.2)	21 (3.6)	31 (5.3)	52 (8.8)
RSV	7 (1.2)	103 (17.5)	110 (18.7)	3 (0.5)	7 (1.2)	10 (1.7)
Adenovirus	2 (0.3)	26 (4.4)	28 (4.7)	1 (0.1)	0	1 (0.16)
Human metapneumovirus	2 (0.3)	13 (2.2)	15 (2.5)	2 (0.3)	3 (0.5)	5 (0.8)
Influenza virus A (H1N1) pdm009	1 (0.1)	29 (0.3)	3 (0.5)	7 (1.2)	7 (1.2)	14 (2.4)
Parainfluenza type 3	2 (0.3)	9 (1.5)	11 (1.8)	2 (0.3)	3 (0.5)	5 (0.8)
Influenza virus A/H3	2 (0.3)	3 (0.5)	5 (0.8)	5 (0.8)	4 (0.7)	9 (1.5)
Parainfluenza type 1	0	5 (0.8)	5 (0.8)	0	2 (0.3)	2 (0.3)
Influenza virus B	0	1 (0.1)	1 (0.1)	1 (0.1)	5 (0.8)	6 (1)
Parainfluenza type 2	1 (0.1)	5 (0.8)	6 (1)	0	0	0
Bacteria^b^	7 (1.2)	59 (10)	66 (11.2)	31 (5.2)	31 (5.2)	62 (10.5)
*Mycoplasma pneumoniae*	1 (0.1)	19 (3.2)	20 (3.4)	9 (1.5)	12 (2.0)	21 (3.5)
*Chlamydophila pneumoniae*	1 (0.1)	14 (2.4)	15 (2.5)	2 (0.3)	3 (0.5)	5 (0.8)
*Haemophilus influenzae*	1 (0.1)	8 (1.3)	9 (1.5)	3 (0.5)	4 (0.7)	7 (1.2)
*Moraxella catarrhalis*	1 (0.1)	11 (1.8)	12 (20.3)	1 (0.1)	1 (0.1)	2 (0.3)
*Klebsiella pneumoniae*	2 (0.3)	4 (0.7)	6 (1)	2 (0.3)	2 (0.3)	49 (0.7)
*Streptococcus pneumoniae*	0	1 (0.1)	1 (0.1)	5 (0.8)	4 (0.7)	9 (1.5)
*Staphylococcus aureus*	1 (0.1)	0	1 (0.1)	3 (0.5)	3 (0.5)	6 (1)
*Acinetobacter baumannii*	0	0	0	3 (0.5)	0	2 (0.3)
*Escherichia coli*	0	2 (0.3)	2 (0.3)	0	0	0
*Burkholderia pseudomallei*	0	0	0	2 (0.3)	0	2 (0.3)
*Acinetobacter calcoaceticus*	0	0	0	1 (0.1)	0	1 (0.1)
*Legionella spp.*	0	0	0	0	1 (0.1)	1 (0.1)
*Pseudomonas aeruginosa*	0	0	0	0	1 (0.1)	1 (0.1)
Mixed detection	7 (1.2)	142 (24.1)	149 (25.3)	42 (7.1)	34 (5.8)	76 (12.9)
Mixed viral detection	1 (0.1)	9 (1.5)	10 (1.7)	1 (0.1)	1 (0.1)	2 (0.3)
Mixed bacterial detection	1 (0.1)	25 (4.2)	26 (4.4)	26 (4.4)	15 (2.5)	41 (6.9)
Mixed viral and bacterial detection	5 (0.8)	108 (18.3)	113 (19.2)	15 (2.5)	18 (3)	33 (5.6)

^a^Single viral pathogen identified

^b^Single bacterial pathogen identified

Among children aged < 5 years, RSV was the most common single pathogen detected (110, 18.7%), followed by adenovirus (28, 4.8%) and parainfluenza type 3 (11, 0.5%). For patients ≥5 years, *M. pneumoniae* was found in 21 (3.6%) cases, followed by influenza A (H1N1) pdm09 (14, 2.4%) and RSV (10, 1.7%) (Table 2). No pathogen was detected in 383 (39.4%) of specimens tested. Only 13 of 220 fatal cases had lung biopsy performed, and five (38.5%) had pathogens identified from lung tissues by PCR techniques: parainfluenza virus type 3 (1), *C. pneumoniae* (1), *Acinetobacter calcoaceticus* (1), *K. pneumoniae* and *A. lwoffii* (1), *A. baumannii* and influenza A (H1N1) pdm09 (1) all results from lung biopsy did not have similar PCR results on respiratory specimens.

Of the 337 cases where blood for culture was collected and incubated at the hospitals, only 27 cases had positive result and in six cases these blood culture results matched with the result of the PCR testing from NIH of respiratory specimens; *S. pneumoniae* (3), *A. baumannii* (1), *K. pneumoniae* (1) and *B pseudomallei* (1).

From 589 cases that had positive laboratory results, 485 (82.3%) cases were reviewed by the severe pneumonia clinical network; 412 (69.9%) met the severe pneumonia case definition and consensus was reached on a likely causative agent in 406 (68.9%) (Table 3). Bacterial infection was more common among fatal cases while viral infection was more common among non-fatal cases. Since MERS-CoV testing was initiated in September 2012, no cases have been identified among the 767 patients tested.

**Table 3:** Etiology of severe pneumonia cases reported to the severe pneumonia surveillance system based on case reviews by the Clinical Network, Thailand 2010–2015

Findings of review cases with positive laboratory result that met SevPn case definition	Age < 5 year			Age > 5 years		
	Fatal cases (%)	Non-fatal cases (%)	Overall (%)	Fatal cases (%)	Non-fatal cases (%)	Overall (%)
	*N* = 14	*N* = 268	*N* = 282	*N* = 53	*N* = 77	*N* = 130
Virus^a^	6 (42.8)	114 (42.5)	120 (42.5)	13 (24.5)	29 (37.6)	42 (32.3)
RSV	1 (7.1)	67 (25.0)	68 (24.1)	0	8 (10.4)	8 (6.1)
Adenovirus	1 (7.1)	18 (6.7)	19 (6.7)	1 (1.9)	1 (1.3)	2 (1.5)
Human metapneumovirus	1 (7.1)	13 (4.8)	14 (4.9)	0	3 (3.9)	3 (2.3)
Influenza virus A (H1N1) pdm009	1 (7.1)	2 (0.7)	3 (1.1)	6 (11.3)	6 (7.8)	12 (9.2)
Parainfluenza type 3	1 (7.1)	5 (1.8)	6 (2.1)	1 (1.9)	4 (5.2)	5 (3.8)
Influenza virus A/H3	0	1 (0.3)	1 (.3)	4 (7.5)	3 (3.9)	7 (5.4)
Parainfluenza type 1	0	3 (1.1)	3 (1.1)	0	3 (3.9)	3 (2.3)
Influenza virus B	1 (7.1)	2 (0.7)	3 (1.1)	1 (1.9)	1 (1.3)	2 (1.5)
Parainfluenza type 2	0	3 (1.1)	3 (1.1)	0	0	0
Bacteria^b^	3 (21.4)	41 (15.3)	45 (15.9)	23 (43.4)	18 (23.4)	41 (31.5)
*Mycoplasma pneumoniae*	1 (7.1)	9 (3.3)	10 (3.5)	3 (5.6)	6 (7.8)	9 (6.9)
*Chlamydophila pneumoniae*	0	8 (2.9)	8 (2.8)	0	0	0
*Chryseobacterium meningosepticum*	0	1 (0.3)	1 (0.3)	0	0	0
*Haemophilus influenzae*	0	7 (2.6)	7 (2.5)	1 (1.9)	1 (1.3)	2 (1.5)
*Moraxella catarrhalis*	0	9 (3.3)	9 (3.2)	1 (1.9)	1 (1.3)	2 (1.5)
*Klebsiella pneumoniae*	2 (14.2)	2 (0.7)	4 (1.4)	4 (7.5)	3 (3.9)	7 (5.4)
*Streptococcus pneumoniae*	0	2 (0.7)	2 (0.7)	6 (11.3)	1 (1.3)	7 (5.4)
*Staphylococcus aureus*	0	1 (0.3)	1 (0.3)	2 (3.8)	3 (3.9)	5 (3.8)
*Acinetobacter baumannii*	0	0	0	1 (1.9)	2 (2.6)	3 (2.3)
*Escherichia coli*	0	3 (1.1)	3 (1.1)	2 (3.8)	0	2 (1.5)
*Burkholderia pseudomallei*	0	0	0	2 (3.8)	0	2 (1.5)
*Acinetobacter calcoaceticus*	0	0	0	1 (1.9)	0	1 (0.7)
*Legionella spp.*	0	0	0	0	0	0
*Pseudomonas aeruginosa*	0	0	0	0	1 (1.3)	1 (0.7)
Mixed detection	5 (35.7)	112 (41.8)	117 (41.4)	15 (28.3)	26 (33.7)	41 (31.5)
Mixed viral detection	0	3 (1.1)	3 (1.1)	2 (3.8)	1 (1.3)	3 (2.3)
Mixed bacterial detection	1 (7.1)	15 (5.6)	16 (5.7)	5 (9.4)	10 (12.9)	15 (11.5)
Mixed viral and bacterial detection	4 (28.6)	94 (35.1)	98 (34.7)	8 (15.1)	15 (19.4)	23 (17.7)
Inconclusive	0	0	0	2 (3.8)	4 (5.2)	6 (4.6)

^a^Single viral pathogen identified

^b^Single bacterial pathogen identified

### Surveillance performance

Of 1101 cases identified by surveillance audit who met the SevPn case definition based on chart review, 158 had been reported to the system for a sensitivity of 14.4% (Table 4). The sensitivity in 2011–2012 was 6.5%, increasing to 18.1% in 2013–2014. Case reporting increased in all regions between the two periods. Sensitivity of the system was highest in the southern region and lowest in the northeastern region during both audits. Between 2011 and 2014 the number of cases reported to the system steadily increased from an average of nine cases a month in 2011 to 16 cases a month in 2014.

**Table 4:** Surveillance audit results for severe pneumonia enhanced surveillance network by region and time period, Thailand 2011–2014

Surveillance region	Severe pneumonia cases reported and detected by chart review								
	2011–2012			2013–2014			Total		
	Reported cases	Cases from chart review	% of cases reported	Reported cases	Cases from chart review	% of cases reported	Reported cases	Cases from chart review	% of cases reported
North	12	151	7.9	51	402	12.5	63	553	11.3
Northeast	0	61	0	2	39	5.1	2	100	2.0
East	4	38	10.5	18	81	22.2	22	119	18.4
Central	1	68	1.2	34	117	29.1	35	185	18.9
South	6	34	17.6	22	66	33.3	28	100	28.0

## Discussion

Enhanced surveillance for severe pneumonia was implemented in Thailand in response to a need to improve identification of the causes of unexplained respiratory deaths and critical illnesses [8, 9]. During 5 years of surveillance, the system strengthened the overall ability of the Thailand MOPH to identify pathogens causing severe pneumonia and demonstrated the significance of RSV as a cause of fatal and non-fatal pneumonia cases, in both adults and children.

Additional benefits of the system included improving the investigation of severe pneumonia cases through the use of standardized guidelines, although surveillance sensitivity, while improved over time, remained low. Enhanced surveillance captured just over 6% of eligible cases in 2012, increasing to 18% by 2014. The increased sensitivity over time may have resulted from identification of dedicated hospital focal persons, laboratorians and the clinicians having a better understanding of the system, ongoing efforts to sensitize clinicians to the importance of the surveillance, or increased awareness from concern over possible MERS-CoV importation.

The SevPn clinical network produced a number of guidelines for severe pneumonia case notification and investigation that have been used throughout Thailand. The case and real-time laboratory results reporting in the online severe pneumonia database allowed for expedited results availability for pathogen identification as soon as 24 h after specimen arrival at NIH, helping clinicians make a definitive diagnosis and improving clinician buy-in for the system. The laboratory diagnostic testing platform and reporting algorithm successfully expanded the range of pneumonia pathogens able to be microbiologically confirmed, including newly emerging pathogens such as MERS-CoV. The system was established in 2010 and proved an important sentinel surveillance platform when MERS-CoV emerged in 2012; the majority of specimens tested for MERS-CoV to date in Thailand have come from the SevPn network.

Throughout the surveillance period, a pathogen was detected in over half of the reported cases. In addition to increasing the number of cases with an identified etiology, the SevPn surveillance has led to a valuable specimen bank of stored specimens that could be used for retrospective testing for new pathogens in the future when diagnostic methods become available.

Guidelines for notification and investigation of severe pneumonia created by this system were modified for use in Thailand’s severe acute respiratory infection surveillance, and post mortem examination guidelines developed by the pathology network for this surveillance system informed procedures for a sub-study of the Pneumonia Etiology Research for Child Health (PERCH) study [16–18]. These post-mortem examination guidelines have frequently been used as a training tool for non-pathologist health personnel to strengthen their capacity to perform lung biopsy in fatal pneumonia cases and also shared with other countries, including Cambodia during a hand, foot, and mouth disease outbreak in 2012 [19].

Despite improvements over time, the findings from the SevPn surveillance system have several important limitations: (1) Case reporting was passive. We documented vast under-reporting through surveillance audits with 86% of potential cases identified through chart review not reported to the SevPn system. Low reporting and variability by region make it difficult to know if etiologic findings were representative of all severe pneumonia cases in Thailand. (2) Post-mortem lung biopsy specimens were infrequently submitted, which might have resulted from a lack of pathologists in most hospitals, challenges in obtaining consent, and limited space to conduct the procedure. (3) Despite an extensive testing algorithm, a pathogen was not detected in nearly 40% of cases with specimens submitted, similar to what has been seen in other pneumonia etiology studies [9, 20, 21]. Detection of pathogens from respiratory specimens does not confirm etiology, especially for bacteria that commonly colonize the upper respiratory tract like *S. pneumoniae* and *H. influenzae.* Collection of lower respiratory specimens through endotracheal tubes helped reduce this challenge, but contamination with upper respiratory flora likely occurred; further, patients who died before intubation had only upper respiratory specimens available. Methods used in this enhanced surveillance could be improved if resources allowed for strengthening the networks, in particular the clinical network and regional laboratory network. In order to increase the number and quality of specimens obtained from fatal cases, further training on obtaining consent and performing post-mortem percutaneous transthoracic needle biopsy would likely be beneficial.

## Conclusion

Developing the capacity for enhanced surveillance for severe pneumonia, which can be used to describe the prevalence of known as well as new pathogens, has been important to strengthen Thailand MOPH’s preparedness and rapid response to emerging respiratory pathogens. Despite its low sensitivity, the SevPn surveillance system has built a network and platform that has bolstered Thailand’s ability to detect emerging infectious diseases. This capacity was demonstrated with the quick addition of MERS-CoV to the testing algorithm in 2012. This project has enhanced the capacity of Thailand MOPH to more rapidly identify causes of severe pneumonia, which will contribute to more rapid detection and control of public health threats and thereby enhance global health security.

Although not all components of the surveillance system will continue after 2015, the severe and fatal pneumonia case notification and investigation guidelines and the post-mortem percutaneous transthoracic needle biopsy guidelines remain useful tools that clinicians, laboratory personnel and epidemiologists can employ during future outbreaks of severe respiratory illness of unknown etiology. The result of the 5 years data collection can help establishment of baseline disease burden estimates or trends for monitoring impact of new potential vaccines, especially for RSV; and facilitate other improved pathogen-specific disease control efforts. The availability of additional diagnostic assays and methods to more fully identify potential pneumonia etiologies for emerging and re-emerging pathogens would be beneficial to test banked specimens from this project as well as in future severe respiratory disease outbreaks [13, 22–24]. Incorporating elements of this enhanced surveillance into existing routine disease surveillance can help the Thailand MOPH strengthen preparedness and rapid response capabilities for infectious disease threats.

## Abbreviations

AI: Avian influenza
BOE: Bureau of Epidemiology
CAP: Community-acquired pneumonia
CDC: Centers for Disease Control and Prevention
MERS-coV: Middle East respiratory syndrome coronavirus
MOPH: Ministry of Public Health
NIH: National Institute for Health
PERCH: Pneumonia etiology research for child health
rRT-PCR: real-time reverse transcription polymerase chain reaction
RSV: Respiratory Syncytial virus
SARS: Severe acute respiratory syndrome
SevPn: Severe and fatal pneumonia
SRRT: Surveillance and Rapid Response Team
VTM: Viral transport media
